# 累及胸壁的胸部肿瘤的外科治疗——胸壁切除与重建（附12例报告）

**DOI:** 10.3779/j.issn.1009-3419.2012.02.05

**Published:** 2012-02-20

**Authors:** 少华 马, 潞艳 申, 森恺 李, 晓天 师, 震 梁, 克能 陈

**Affiliations:** 1 100142 北京，北京大学肿瘤医院暨恶性肿瘤发病机制及转化研究教育部重点实验室胸外一科 Key Laboratory of Carcinogenesis and Translational Research (Ministry of Education), Department of Thoracic Surgery Ⅰ, Peking University School of Oncology, Beijing Cancer Hospital & Institute, Beijing 100142, China; 2 100144 北京，中国医学科学院整形外科医院 Plastic Surgery Hospital (Institute), Peking Union Medical College, Beijing 100144, China; 3 455001 安阳，安阳肿瘤医院胸外科 3 Department of Thoracic Surgery, Anyang Cancer Hospital, Anyang 455001, China

**Keywords:** 胸部肿瘤, 胸壁, 切除, 整形, Thoracic neoplasms, Thoracic wall, Resection, Reconstruction

## Abstract

**背景与目的:**

胸部肿瘤累及胸壁是临床常见事件，若无远处转移，完整切除受累胸壁仍可获得良好疗效。本文结合12例肿瘤患者胸壁切除与重建（chest wall resection and reconstruction, CWRR）的经验就重建人工材料、软组织覆盖等方面作一介绍，并强调切除外科与重建外科合作的重要性。

**方法:**

总结2005年10月-2011年4月北京大学肿瘤医院胸外一科和重建外科共同参与的CWRR 12例，详细复习自确诊至今的诊治全过程，包括术前治疗、手术方式、切除范围、重建方式，主要的局部及全身并发症及生存情况。

**结果:**

12例均为根治性手术，均行骨性胸壁切除，切除后骨性胸壁缺损为25 cm^2^-700 cm^2^，胸壁软组织缺损为56 cm^2^-400 cm^2^。骨性胸壁修补材料采用聚丙烯单丝网片（polypropylene mesh），软组织修复采用转移肌瓣、转移肌皮瓣及大网膜瓣。术后1例发生呼吸衰竭，呼吸机辅助通气1个月后痊愈，余11例均无并发症，全组12例至今全部存活。

**结论:**

只有切除外科和重建外科同时参与才能完成符合肿瘤原则的复杂CWRR。由切除外科主导、重建外科协助、了解并熟悉重建材料及胸壁软组织重建，是达到手术根治性及保证远期生存的关键。

## Abstract (en)

**Background and objective:**

Tumor involving the chest wall is a common clinical event, and if there are no distant metastases, complete resection of tumor and involved chest wall can give excellent results. The aim of this study is to report experience with chest wall resection and reconstruction (CWRR) for 12 patients who suffered thoracic malignant tumor involving chest wall, including the artificial materials used for reconstruction, soft tissue coverage, and our multidisciplinary CWRR approach.

**Methods:**

All characteristics of 12 cases of CWRR from Oct 2005 to Apr 2011 were reviewed, including preoperative treatment, surgical approach, resection range, reconstruction methods, the local and systematic complications and postoperative survival.

**Results:**

All 12 of these patients underwent radical resection and bony chest wall resection, with resultant bony chest wall defects ranging from 25 cm^2^ to 700 cm^2^, soft tissue defects of 56 cm^2^ to 400 cm^2^. The bony chest wall was reconstructed using polypropylene mesh, and repair of the soft tissue was carried out using the shifting muscle flaps, myocutaneous flaps and omental flaps. There was only one significant complication in these 12 cases where 1 case suffered respiratory failure and needed mechanical ventilation but recovered one month later. All 12 patients have survived to the end point of follow up.

**Conclusion:**

Only thoracic surgery and reconstructive surgery work together can complete the complex CWRR which according the tumor discipline. Thoracic surgeons as the leader and reconstructive surgeons as the subsidiary and be familiar with reconstruction materials of bony chest wall and appropriate choice of soft tissue coverage is the key to achieve radical surgery and to ensure long-term survival.

胸壁原发性肿瘤和胸部肿瘤侵犯胸壁是胸外科常见的临床情况，需行胸壁切除，对胸外科医生来讲切除胸壁并非难事。然而，胸壁的完整性是维持人体正常呼吸循环生理解剖的重要组成部分，切除后联合整形外科，需选用合适的人工材料重建胸壁以恢复其解剖和生理，并选用合适的软组织覆盖以保护人工材料以便最终愈合，从而达到根治肿瘤，改善疗效的目的。现将北京大学肿瘤医院的12例胸壁切除与重建（chest wall resection and reconstruction, CWRR）治疗经验总结如下。

## 1 患者与方法

2005年10月-2011年4月北京大学肿瘤医院胸外一科和重建外科共同参与的CWRR 12例（表 1），其中男性8例，女性4例，年龄37岁-78岁，包括左侧胸壁软骨肉瘤术后复发侵犯第6、7、8肋骨，前锯肌，左肺下叶，心包及膈肌1例；右乳腺癌术后放疗局部皮肤破溃30年，恶变为疣状癌，侵犯双侧第2、3、4、5肋软骨，部分胸骨，右侧胸大肌及心包1例；左胸壁恶性纤维细胞瘤术后复发，侵犯前锯肌及左第4、5、6肋骨1例；右肺上叶腺癌侵犯右前第3、4肋骨及前锯肌1例；左乳腺癌术后3.5年局部胸壁复发侵犯左胸大肌，胸小肌，左第2、3、4肋软肋骨及左侧部分胸骨1例；前纵隔鳞癌术后复发侵犯右侧第4、5前肋，右侧部分胸骨，胸小肌及胸大肌1例；左肺上叶癌术后8个月，局部胸壁复发，侵犯左第3、4肋骨及前锯肌1例；胸壁孤立性转移癌侵犯左后第8肋骨，前锯肌及下后锯肌1例；左肺上叶腺癌侵犯第1、2前肋及胸小肌1例；右肺上叶鳞癌侵犯右前第2肋骨及胸小肌1例；左肺上叶腺癌侵犯左第4后肋1例；前纵隔弥漫大B细胞淋巴瘤侵犯左侧第4、5肋骨及下1/2胸骨1例。

**1:** 12例胸壁切除与重建患者一般资料 General data of 12 patients with CWRR

No.	Gender	Age	Diagnosis	Tumor diameter (mm)	Bony chest wall invading	Soft tissue invading	Neo-adjuvant chemo	Adjuvant chemo
1	Male	68	Chondrosarcoma recurrence of left chest wall	250×280	6^th^, 7^th^, 8^th^ rib	Serratus anterior	No	No
2	Female	76	Local skin ulceration malignant transform to verrucous carcinoma after mastectomy	80×50	Bilateral 2^nd^, 3^rd^, 4^th^, 5^th^ costal cartilage and partial of stemum	Pectoralis major	No	No
3	Female	37	Recurrence malignant fiber cell tumor of left chest wall	110×90	4^th^, 5^th^, 6^th^ rib	Serratus anterior	No	No
4	Male	73	Adenocarcinoma of left upper lobe lung	25×25	3^rd^, 4^th^ rib	Serratus anterior	No	TP×4
5	Female	53	Local recurrence of left chest wall after radical mastectomy	30×30	2^nd^, 3^rd^, 4^th^ rib and partial of sternum	Pectoralis major, pectoralis minor	No	No
6	Male	78	Anterior mediastinal recurrence squamous cell carcinoma	70×52	4^th^, 5^th^ rib and right partial sternum	Pectoralis major, pectoralis minor	TP×3	No
7	Male	60	Recurrence squamous cell carcinoma after pulmonary lobectomy of left upper lobe	50×40	3^rd^, 4^th^ rib	Serratus anterior	No	No
8	Male	53	Isolation metastatic carcinoma of left chest wall	55×45	8^th^ posterior rib	Serratus anterior and serratus posterior inferior muscle	No	TP×2
9	Male	53	Adenocarcinoma of left upper lobe lung	30×30	1^st^, 2^nd^ anterior rib	Pectoralis minor muscle	TP×1	No
10	Male	74	Squamous cell carcinoma of right upper lobe lung	75×60	2^nd^ anterior rib	Pectoralis minor muscle	TP×2	No
11	Female	56	Adenocarcinoma of left upper lobe lung	30×30	4^th^ posterior rib	No	TP×2	TP×2
12	Male	39	Diffuse large B-cell lymphoma of anterior mediastinal	50×50	Left 4^th^, 5^th^ rib and partial of stenum	No	No	No
CWRR: chest wall resection and reconstruction; Chemo: chemotherapy								

12例患者术前均行胸部增强计算机断层显像（computerized tomography, CT）检查，2011年以前手术的7例患者均行常规分期检查，如颈部及腹部超声、骨扫描、头颅核磁等，2011年手术的5例患者除常规分期检查外，加行正电子发射计算机断层显像（positron emission tomography-computerized tomography, PET-CT）检查，其中1例在2周期新辅助化疗后复查PET-CT。术前对心肺功能做详细评估，包括心脏彩超、肺功能及动脉血气分析。所有患者术前均与整形外科医生会诊，设计切口，骨性胸壁及软组织切除范围及软组织修补方式。

## 2 结果

2011年以前手术的7例患者术前常规肿瘤分期检查均未见远处转移及肿瘤淋巴引流区域明显肿大淋巴结。2011年手术的5例患者除常规分期检查外加行PET-CT检查，除肿瘤本身外均未见全身其它部位及肿瘤淋巴引流区域高代谢病灶。12例术后病理均证实无引流区域淋巴结转移。5例行PET-CT检查中的1例患肺癌者行新辅助化疗，在化疗后复查PET-CT，其标准摄取值（standard uptake value, SUV）明显下降，且此5例术前均加做了CT三围胸壁骨成像（图 1），明确显示了骨性胸壁受累情况，与术后病理结果吻合。12例均行根治性切除（表 2）。肿瘤直径25 mm×25 mm-250 mm×280 mm，其中1例肿瘤导致皮损面积达200 mm×200 mm（图 2）。所有切口均根据肿瘤生长部位及范围来决定，均为不规则切口，切口总长度从15 cm-90 cm不等（图 2）。切除后胸壁软组织缺损56 cm^2^-400 cm^2^；同时切除骨性胸壁12例，骨性胸壁缺损25 cm^2^-700 cm^2^，骨性胸壁修补材料采用聚丙烯单丝网片（图 3），软组织修复采用转移肌瓣、转移肌皮瓣或大网膜转移瓣，其中胸大肌转移肌瓣4例（图 3），背阔肌转移肌瓣3例（图 4），胸大肌及背阔肌联合转移肌瓣1例，腹直肌肌皮瓣1例（图 5），大网膜转移瓣1例（图 6）。1例由于为感染切口未行人工材料修补，而采用了广泛游离的皮瓣修复（图 2）。其修复方法为以肿瘤为中心的“X”形切口，皮瓣游离范围上达双侧锁骨水平，下达髂前上棘，外侧达双侧腋后线，此例术后呼吸衰竭（限制性通气障碍），呼吸机辅助通气1个月后痊愈。全组中另有1例系后胸壁切除，有肩胛骨覆盖且未累及胸部软组织而只切除胸壁未作真正意义上的修复。12例患者随访至2011年5月15日，全部存活，其中1例左侧胸壁恶性纤维细胞瘤行CWRR术后43个月发生肺转移，再行肺叶部分切除术。

**1:**
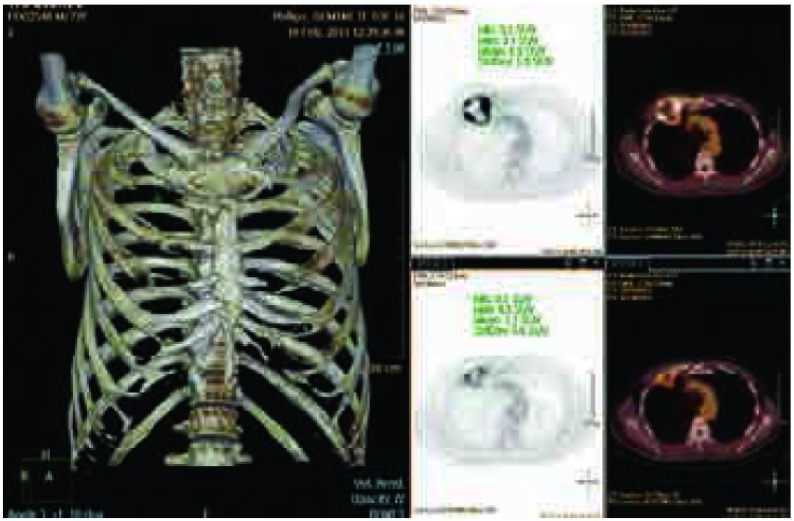
病例10，男性，74岁，右肺上叶癌，侵犯右第2前肋及胸小肌，实施新辅助化疗2周期后手术。术前CT三位骨重建明确显示右第2前肋受累，与化疗前PET比较，局部病灶最大SUV值由10.8降至7.7。 Case 10, 74-year old male, with lung cancer of right upper lobe invading 2^nd^ rib and pectoralis minor muscle. Treated with 2 cycles of neo-adjuvant chemotherapy followed by surgery. Three-dimensional CT synthetic image shows tumor invading 2^nd^ rib, compare with the first PET/CT prior to chemotherapy (top right). SUV decreased from 10.8 to 7.7. SUV: standard uptake value.

**2:** 12例胸壁切除及成形患者手术情况 Operation features of 12 patients treated with CWRR

No.	Organs resection	Defect of bony chest wall (mm)	Defect of soft tissue (mm)	Material of bony chest wall reconstruction	Coverage	Operation time	Survival time (month)
1	Partial of left lower lobe, partial of pericardium, partial of diaphragm, 6^th^, 7^th^ and 8^th^ rib	250 ×280	150 ×150	Marlex	Left latissimus dorsi musclar flap	2005.10.15	66
2	Bilateral 2^nd^, 3^rd^, 4^th^, 5^th^ costal cartilage, partial of sternum, partial of left upper lobe, partial of pericardium	100 ×80	200 ×200	No	Widely dissociative skin flaps	2007.9.7	43
3	Left 4^th^, 5^th^, 6^th^ rib, partial of left lower lobe	140 ×120	140 ×120	Marlex	Left latissimus dorsi muscle	2007.9.9	43
4	Right upper lobe of lung, 3^rd^, 4^th^ rib, partial of serratus anterior muscle	70 ×50	80 ×70	Polypropylene monofilament	Right pectoralis major muscle flap	2009.2.4	26
5	Left 2^nd^, 3^rd^, 4^th^ costal cartilage, partial left sternum	100 ×60	110 ×70	Polypropylene monofilament	Right pectoralis major muscle flap	2009.5.25	23
6	Right 3^rd^, 4^th^, 5^th^, 6^th^ rib, partial of right sternum	140 ×120	200 ×150	Polypropylene monofilament	Left rectus abdominis muscleflap	2009.10.28	18
7	Left 2^nd^, 3^rd^, 4^th^, 5^th^ rib, partial of serratus anterior muscle	120 ×110	120 ×120	Polypropylene monofilament	Left pectoralis major muscle flap and latissimus dorsi flap	2010.5.18	11
8	Left 7^th^, 8^th^, 9^th^ rib, partial of serratus anterior muscle, partial of serratus posterior inferior muscle	100 ×65	12 0 ×70	Polypropylene monofilament	Latissimus dorsi flap	2011.2.15	2
9	Left upper lobe of lung, 1^st^, 2^nd^, 3^rd^ rib, partial pectoralis minor muscle	100 ×100	120 ×120	Polypropylene monofilament	Pectoralis major muscle flap	2011.4.1	1
10	Right upper lobe of lung, 1^st^, 2^nd^, 3^rd^ rib, partial pectoralis minor muscle	100 ×100	120 ×120	Polypropylene monofilament	Pectoralis major muscle flap	2011.4.9	1
11	Left upper lobe of lung, 4^th^, 5^th^ rib	50 ×50	0	Scapula	No	2011.4.10	1
12	Left upper lobe of lung, 3^rd^, 4^th^, 5^th^ rib, partial of sternum	120 ×80	150 ×100	Polypropylene monofilament	Great omental flap	2011.4.29	1

**2:**
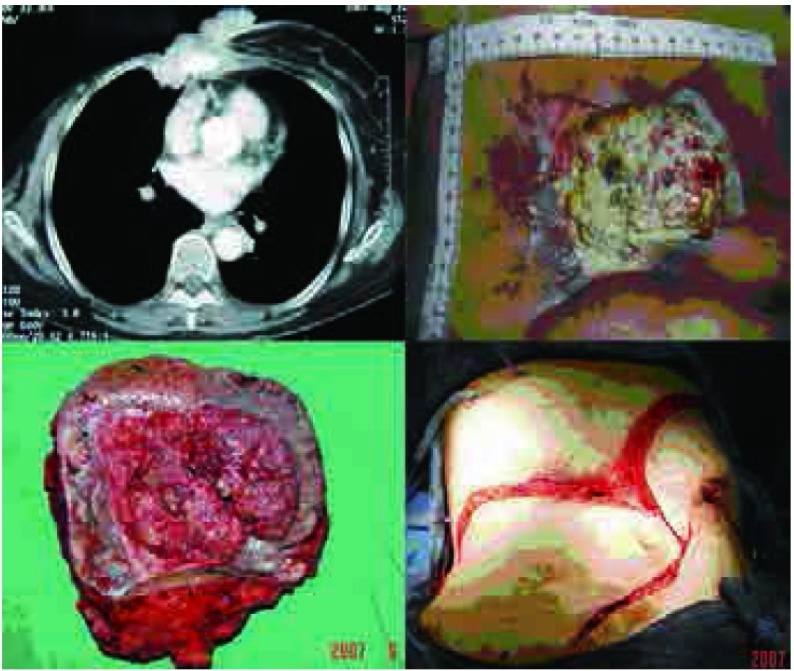
病例2，女性，76岁，30年前行右乳腺癌改良根治术，术后放疗致皮肤溃疡经久不愈，切除肿瘤80 mm×50 mm，骨性胸壁缺损为100 mm×80 mm，皮损面积达到200 mm×200 mm，因感染，未行人工材料修补，仅广泛游离左侧乳腺及上腹部皮瓣修补软组织。 Case 2, 76-year old female, underwent radical mastectomy followed by radiotherapy. Local skin ulcer prolong for 30 years. She underwent CWRR, and the tumor was 80 mm×50 mm, the defect of bony chest wall was 100 mm×80 mm, and the soft tissue defect reached to 200 mm×200 mm. Due to infection, synthetic materials were not used, widely dissociative skin flaps were used to repair the large chest wall defect.

**3:**
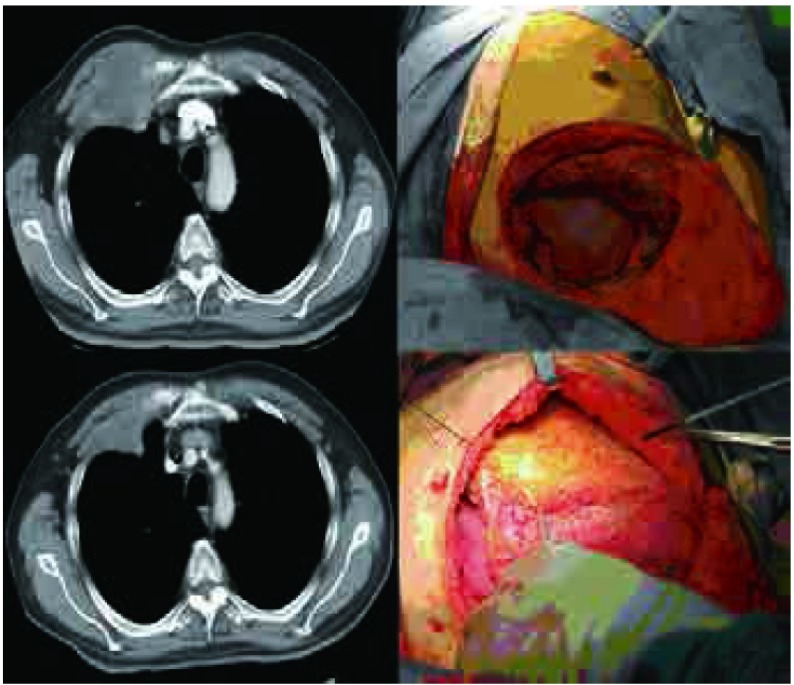
病例10，男性，74岁，右肺上叶癌，侵犯右第2前肋及胸小肌，实施新辅助化疗2周期后，实施右肺上叶+第1、2、3部分肋骨+部分胸小肌切除，骨性胸壁缺损达100 mm×100 mm，采用聚丙烯单丝网片修补，软组织修复采用胸大肌转移肌瓣。 Case 10, 74-year old male Lung cancer of right upper lobe, with invasion of 2^nd^ rib and pectoralis minor muscle. Treated with 2 cycles of neo-adjuvant chemotherapy followed by surgery consisting of right upper lobectomy, the 1^st^, 2^nd^ and 3^rd^ ribs and partial pectoralis minor muscle resection. The bony chest wall defect was 100 mm×100 mm, and polypropylene mesh was used for reconstruction and the synthetic material was covered by pectoralis major muscle flap.

**4:**
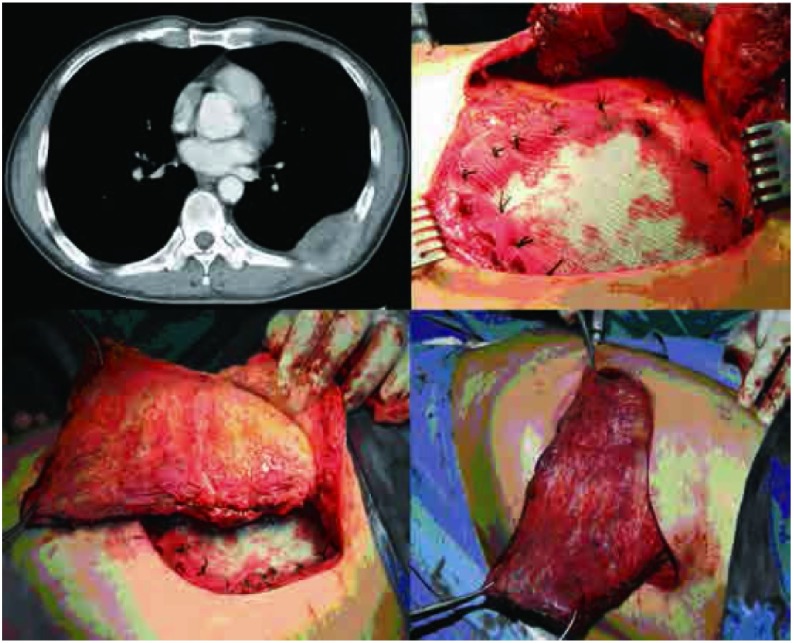
病例8，男性，53岁，左后胸壁单发肿瘤，侵犯左后第8肋骨，实施左后第7、8、9肋骨部分切除，切除后骨性胸壁缺损100 mm×65 mm，采用聚丙烯单丝重建骨性胸壁，背阔肌转移肌瓣修补软组织 Case 8, 53-year old male, isolated left chest wall metastasis involving the posterior left 8^th^ rib, underwent CWRR with resection of left 7^th^, 8^th^, and 9^th^ ribs. The bony chest wall defect was 100 mm×65 mm, and polypropylene mesh was used for to reconstruction, which was covered with latissimus dorsi muscle flap.

**5:**
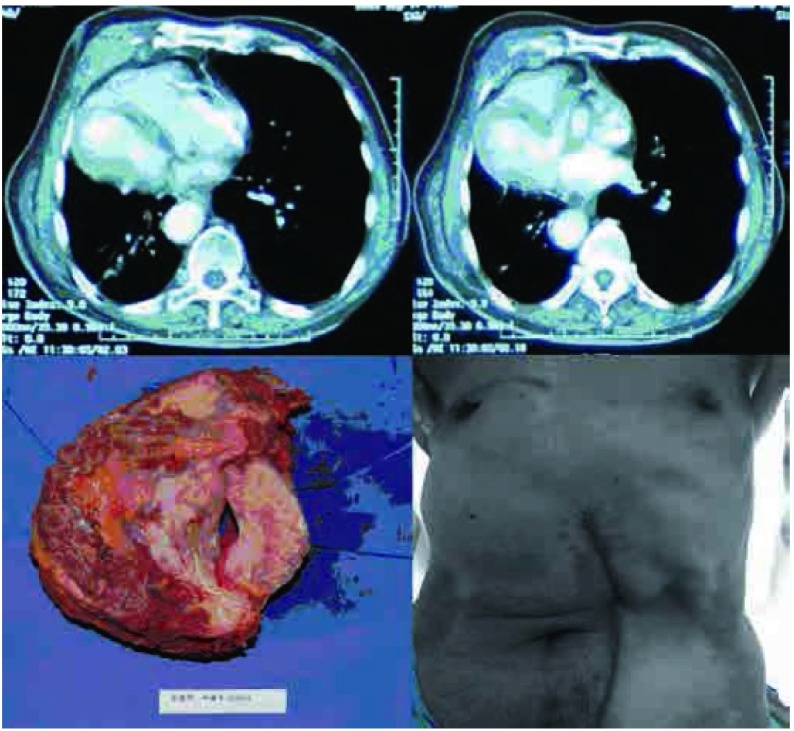
病例6，男性，78岁，前纵隔鳞癌术后复发，侵犯右第4、5肋骨、右侧部分胸骨、胸小肌及胸大肌，实施右第3、4、5、6前肋、右侧部分胸骨、部分胸小肌及部分胸大肌切除，骨性胸壁缺损140 mm×120 mm，软组织缺损200 mm×150 mm，采用左侧腹直肌转移肌瓣修复胸壁软组织缺损。 Case 6, 78-year old male, Anterior mediastinal postoperative recurrence of squamous cell carcinoma, which involved the right 4^th^ and 5^th^ ribs, part of the sternum, and the pectoralis minor and major muscles. Treated with CWRR, with resection of the right 3^rd^, 4^th^, 5^th^ and 6^th^ ribs, partial sternal resection, and partial resection of the pectoralis minor and major muscles. The bony chest wall defect measured 140 mm×120 mm, with a soft tissue defect of 200 mm×150 mm. Polypropylene mesh was used to repair the chest wall defect and a left rectus muscle flap was transferred to cover the artificial material.

**6:**
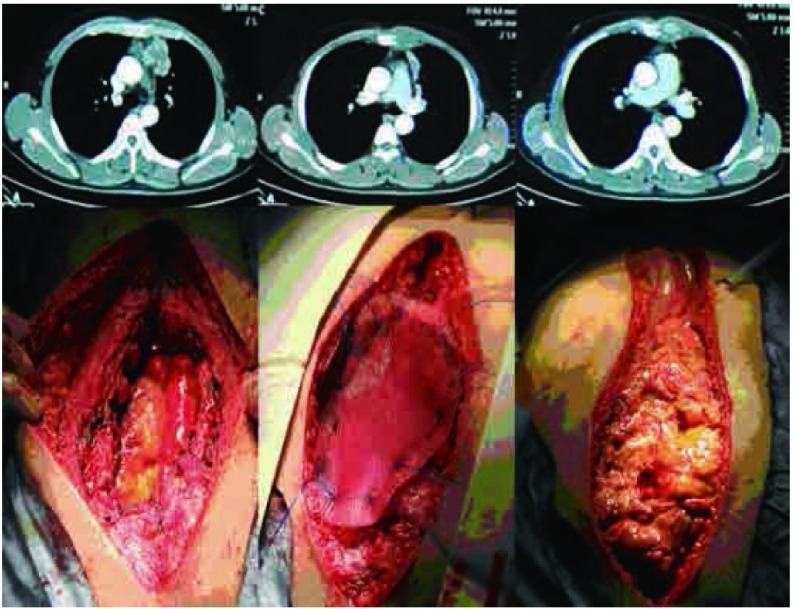
病例12，男性，39岁，前纵隔弥漫大B细胞淋巴瘤，侵犯第4、5肋骨+左侧部分胸骨，实施双侧3、4、5、6肋软骨切除+部分胸骨切除+左肺上叶切除，骨性缺损120 mm×80 mm，采用聚丙烯单丝修补，软组织缺损达150 mm×100 mm，采用大网膜转移瓣修补 Case 12, 39-year old male, with lymphoma of the anterior mediastinum involving the 4^th^ and 5^th^ ribs and part of the sternum. Treated with bilateral 3^rd^, 4^th^, 5^th^ and 6^th^ costal cartilage resection, partial sternal resection and resection of the left upper lung lobe. The bony chest wall defect measured 120 mm×80 mm and soft tissue defect measured 150 mm×100 mm. Polypropylene mesh was used to repair the bony defect followed by use of a greater omental flap to cover the artificial material.

## 3 讨论

### 3.1 肿瘤根治原则是胸壁切除术的前提

胸壁切除与重建的创伤较大，除少数情况以外，保证根治性原则使患者生存获益是该治疗的前提。胸部肿瘤需胸壁切除的情况包括：原发性胸壁骨软组织肿瘤，相邻部位肿瘤侵犯、复发及遗留的创伤并发症，如溃疡、坏死等，孤立的胸壁转移。这一工作主要由胸外科医生完成，包括对原发疾病分期的评估，胸壁切除的范围与硬性胸壁重建的设计等，其中以实施全面分期检查尤为重要。文献^[1]^报道肺癌累及胸壁行CWRR后，N0患者术后5年生存率明显高于N1/N2患者，分别为39.2%和7.1%。乳腺癌侵犯胸壁行CWRR后的5年生存率为39.2%，而复发性乳腺癌行CWRR后中位生存时间可达36个月^[2]^。本组2011年以前手术的7例患者，术后病理证实均未见淋巴结转移，术后均存活，最长1例已存活66个月。常用的术前影像学分期方法以CT、核磁共振等为主，值得一提的是，近年来以PET-CT为主要代表的功能影像学的常规应用更为重要^[3]^。2011年手术的5例患者除传统的分期检查外，均采用了PET-CT检查，5例患者除局部病灶外均未发现远处转移及淋巴结转移，而且与术后病理结果一致。其中的第10例患者新辅助化疗2周期后复查PET-CT提示SUV值降低，肿瘤明显缩小，更为术后的辅助化疗提供了良好的依据。

### 3.2 骨性胸壁的重建是恢复心肺功能的保证

对呼吸功能较好，切除范围较小者，CWRR对呼吸的机械影响可忽略不计，若以软组织瓣覆盖也可在一定程度上克服术后胸壁的反常呼吸^[4-7]^。但对缺损较大者需行骨性胸壁重建。国内尝试过多种稳定性材料，包括自体和人工材料两类，前者因为额外增加手术切口而被淘汰。人工材料如不锈钢、钛合金、透明树酯、玻璃纤维等硬材料难以避免腐蚀、感染，也很少应用^[8]^。即便近年来国内的文献^[9]^报道胸壁重建材料为有机玻璃板、玻璃条、钢丝网、钛板、涤纶、硅橡胶等，但也因为修补材料与组织结合困难，后期常常松动造成组织破坏而逐渐被淘汰。目前，良好的骨性胸壁重建材料需要具备以下几个条件^[9]^：①柔软易放置，顺应性好，且能维持胸壁的强度及美观；②组织反应性小，与组织能够完全融合；③感染风险较低；④机械强度高，以便能够维持很长时间；⑤能够成为潜在早期复发者手术切除的标记。近年流行的Marlex网、polypropylene网等网状材料，其特点为多孔网状，可与组织融合，且融合后顺应性好，更适合胸壁重建^[7, 10]^。本组全部选用聚丙烯单丝网状补片，组织相容性好，顺应性强且维持了一定的胸壁硬度。

### 3.3 术前与整形外科的会诊，多学科会诊是治疗成功的关键

后外侧切口，前外侧切口及正中切口是胸部手术常用和孰知的切口，这不但有利于保护胸壁软组织，减少创伤，也便于肋间进胸，可完成绝大多数的胸内操作，因而为广大的胸外科医生熟知和广泛接受。但胸壁切除与重建往往需要联合切口，甚至是依肿瘤的生长选择不规则切口，因而术前需要与整形外科医生会诊，会诊的目的在于外科医生与整形外科医生共同商议切除范围，切口的难易程度等，从而便于设计修复途径，使胸外科医生了解首选、次选和最后的修复设计，了解各个皮瓣的血运来源，从而达到最大程度的根治性以保证疗效，最好保留重建肌皮瓣的血运以达到最好的修补效果。

CWRR手术要点为骨性胸壁修复及肌瓣/肌皮瓣的转移及应用。应用网状补片材料修补骨性缺损后，需要适当的软组织覆盖，防止皮肤坏死，整形外科医生在其中有重要的作用，如本组第2例患者系乳腺癌改良根治术后放疗致皮肤溃疡经久不愈就诊，因感染胸壁切除后不宜使用人工材料修复，但仍然要维持胸壁的硬度，则需要更厚的软组织皮瓣来修补，遂采用右侧腹直肌及对侧胸大肌联合肌瓣，肌瓣外再以对侧乳腺组织覆盖以完成修复。

总之，胸壁切除与重建在胸部肿瘤有广泛的应用途径，熟知切除的生存获益使广大胸壁受累的患者获得了根治机会，熟知重建材料、肌瓣的进展也为更多的患者提供了治愈的机会。
